# The role of exosomes in immunopathology and potential therapeutic implications

**DOI:** 10.1038/s41423-025-01323-5

**Published:** 2025-07-14

**Authors:** Wenhui Wang, Shuya Qiao, Xianghui Kong, Gensheng Zhang, Zhijian Cai

**Affiliations:** 1 Department of Orthopedics of the Second Affiliated Hospital and Institute of Immunology, Zhejiang University School of Medicine, Hangzhou, China; 2 Liangzhu Laboratory, Zhejiang University School of Medicine, Hangzhou, China; 3 Bone Marrow Transplantation Center of the First Affiliated Hospital and Institute of Immunology, Zhejiang University School of Medicine, Hangzhou, China; 4 Department of Critical Care Medicine of the Second Affiliated Hospital, Zhejiang University School of Medicine, Hangzhou, China

**Keywords:** EVs, Exosomes, Biogenesis, Immunopathology, Tumor, Cargo sorting, Immunology, Cell biology

## Abstract

Extracellular vesicles (EVs), including exosomes, ectosomes, and apoptotic bodies, are released by various cells. Among these subtypes, exosomes have been extensively studied and demonstrated to be crucial mediators of intercellular communication involving multiple physiological and pathological processes. Four primary steps influence the biogenesis of exosomes: generation of early endosomes, formation and maturation of multivesicular bodies (MVBs), MVB and plasma membrane fusion for exosome release, and MVB fusion with lysosomes for degradation. During the formation and maturation of MVBs, the main effector molecules, such as RNAs and proteins, are sorted into exosomes via diverse mechanisms. However, the effector molecules of exosomes are dynamic and reflect cell states in real time. Therefore, exosomes secreted by cells under disease conditions are often pathogenic. This review focuses on recent advances in the understanding of exosome biogenesis and the immunopathological effects of exosomes. In addition, potential strategies to mitigate the pathological effects of exosomes are summarized in this review.

## Introduction

Extracellular vesicles (EVs) mainly consist of exosomes and ectosomes. Exosomes are produced via the endosome pathway, while ectosomes are released by outward budding of the plasma membrane (PM). The diameter of ectosomes varies greatly, ranging from tens of nanometers to several micrometres. The diameter of exosomes is relatively small, ranging from 30 to 150 nm [1]. According to the MISEV2023 guidelines, the direct use of secretion pathway-based terminology such as “exosomes” and “microvesicles” is no longer recommended, as their biogenesis is often difficult to trace and lacks universal applicability. Instead, the guidelines propose classifying EVs into small EVs (sEVs) (diameter <200 nm, corresponding to traditional exosomes and a subset of microvesicles <200 nm) and large EVs (lEVs) (diameter >200 nm, encompassing traditionally defined microvesicles >200 nm and apoptotic bodies) [2]. While most studies using classical ultracentrifugation methods primarily isolate sEVs [3] and since current research on the biogenesis of exosomes is relatively well established [4], this article specifically focuses on recent advances in exosome biogenesis—the term “exosomes” is retained here. However, owing to the lack of specific markers, the EVs isolated in the cited literature are not exclusively composed of exosomes, as they may contain vesicles from multiple biogenetic pathways.

Almost all types of cells can release exosomes. Exosomes are considered ‘platelet dust,’ a byproduct of the body’s natural waste disposal processes and maintain homeostasis for a long period of time [5]. However, they are now widely recognized as critical cellular signaling and intercellular communication tools. Exosomes share a similar molecular composition and structural configuration with their parental cells, effectively serving as functional extensions. Owing to their high degree of topological similarity to cells, exosomes can traverse multiple cell types and physiological barriers, such as the blood‒brain, intestinal, placental, etc. [6–8]. Remarkable accessibility positions exosomes as vital mediators of intercellular communication, bridging central and peripheral communication and cross-organ interactions.

The cargos carried by exosomes include membrane-bound/soluble proteins, nucleic acids, lipids, and metabolites, reflecting their parent cells’ origin and functional specificity. On the one hand, exosomes protect their cargos from degradation and inactivation, ensuring their integrity during delivery to target cells. On the other hand, exosomes facilitate the entry of their cargo and functional integration in recipient cells. These distinctive features position exosomes as pivotal modulators of cell phenotypes and functions, particularly in regulating and reprogramming immune cells under pathological conditions [9]. In this review, we summarize the origin, cargo sorting, and role of exosomes in modulating immune cells across different systems and tumor immunity, with a particular focus on their functions during pathological processes. In addition, we summarize potential strategies for eliminating the pathological effects of exosomes.

## History of exosome discovery

Research on exosomes has been conducted over the past five decades [10]. In 1967, Wolf discovered that minute particulate material could be obtained by ultracentrifugation in fresh plasma from which platelets had been removed. He named those particles ‘platelet dust' [5]. Crawford published additional images of these vesicles containing lipids and contractile proteins, describing them as ‘microparticles’ in 1971 [11]. Two seminal studies published by Harding et al. and Pan et al. in the 1980s marked the expansion of exosome research. These two researchers independently discovered a novel intracellular sorting and trafficking pathway called the exosomal secretory pathway [12, 13]. In brief, they reported that reticulocytes are capable of secreting small vesicles ~50 nm in size during the maturation process. These small vesicles help reticulocytes export transferrin into the extracellular space [12, 13]. A few years later, Johnstone et al. coined the term ‘exosome’ to describe these vesicles, marking a pivotal moment in exosome research [14].

These early studies provided a solid foundation for the subsequent surge of interest over the following years. Since then, with the development of techniques such as molecular biology, nanotechnology, and flow cytometry, the biological functions of exosomes have been gradually explored in greater depth, and research into their roles in health and disease has expanded significantly. Raposo et al. discovered that B lymphocytes can secrete exosome particles containing MHC-II molecules in 1996. These molecules can stimulate the immune response of CD4^+^ T cells [15]. This discovery established the foundation for further research into exosome biology within the context of immunology. Since Lötvall and colleagues reported that exosomes can mediate the transfer of messenger RNA (mRNA) and microRNA (miRNA) between cells in 2007, the ability of exosomes to mediate intercellular communication by transferring a wide range of molecular cargos, including proteins, lipids, mRNAs, miRNAs, and other noncoding RNAs, has attracted increasing attention [16]. These cargos influence the behavior and function of target cells, making exosomes essential messengers in various biological processes. Rothman, Schekman, and Sudhof won the 2013 Nobel Prize in Physiology or Medicine for discovering the regulatory mechanism of intracellular vesicle transport, which elevated exosome research to a new echelon [17–19]. Since then, exosome research has been utilized in various fields, including stem cells, immunity, miRNA, targeted drug delivery, disease diagnosis, and therapy.

## Biogenesis of exosomes

**Fig. 1:**
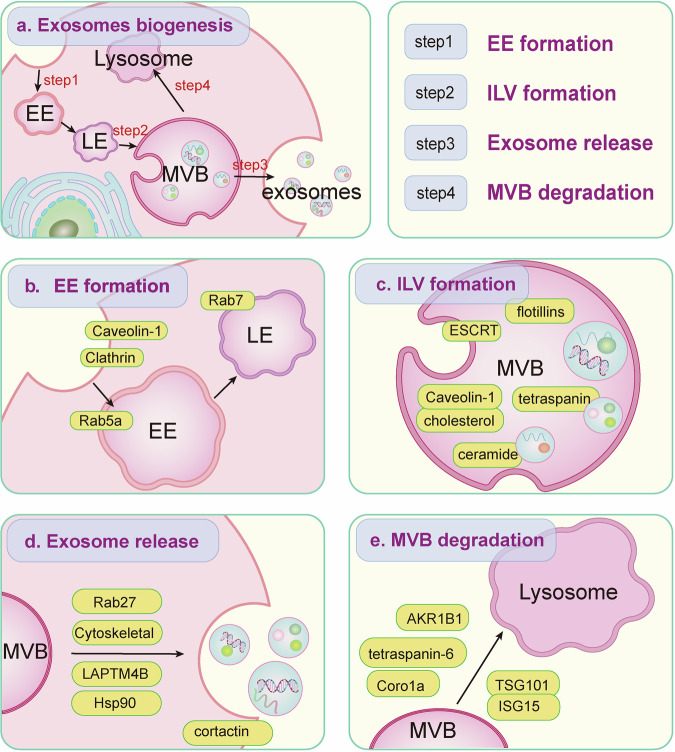
Biogenesis of EVs. **a** Four critical steps of exosome biogenesis. **b** Regulators of EE formation involving exosome origin. **c** Regulators of ILV formation. **d** Regulators of MVB and PM fusion. **e** Regulators of MVB and lysosome fusion

The biogenesis, sorting, and release of different types of exosomes involve a series of finely regulated processes. i) Early endosomes (EEs) form the initial stage of exosome formation; ii) then, EEs mature into late endosomes (LEs) along with inward buds to form multivesicular bodies (MVBs) containing many intraluminal vesicles (ILVs); and iii) finally, the MVBs are transported to and fuse with the PM, leading to the release of exosomes into the extracellular space. The successful completion of these three processes is conducive to exosome biogenesis. iv) If MVBs fuse with lysosomes, they are degraded, inhibiting exosome formation (Fig. 1a). Currently, two main types of exosome biogenesis exist: endosomal sorting complex required for transport (ESCRT)-dependent and ESCRT-independent pathways [20–22]. This classification is based primarily on the second step of exosome generation, namely, the generation of ILVs, which also includes how cargos are sorted into ILVs. Here, we classify exosome biogenesis into the four steps mentioned above and summarize the known mechanisms responsible for the regulation of each step.

## Regulation of EE formation

The initial phase in the formation of exosomes is the inward budding of the PM, which results in the formation of EEs. In theory, effector molecules that regulate the generation of EEs can affect the generation of exosomes (Fig. 1b).

Caveolin-1 is a marker protein for caveolae generation that can invaginate the PM and is involved in the formation of caveolae structures, vesicle transport, and cholesterol homeostasis maintenance. The literature on caveolin-1 and EEs is limited, with most studies concentrating on regulating ILV generation [23, 24]. However, on the basis of the available evidence, it can be hypothesized that caveolin-1 may regulate EE generation by promoting foveal formation. Although there is no direct literature showing that caveolin-1 can affect the release of exosomes by regulating EE generation, there is literature showing that caveolin-1 can be released through exosomes in prostate cancer cells [25].

Clathrin has been demonstrated to play a pivotal role in clathrin-mediated endocytosis [26]. By forming an icosahedral cage shape, clathrin displaces the membrane inward, thereby concentrating cargo into clathrin-coated pits [27]. It has been demonstrated that vesicles containing clathrin can be enriched in EEs and involved in the subsequent PM fusion pathway, which can have a particular effect on exocytosis [28]. In macrophages, enzymes are internalized mainly by clathrin-mediated endocytosis and then trafficked to recycling endosomes. The enzyme is subsequently released in exosomes, facilitated by bridging conduits [29]. These findings suggest the potential role of clathrin-mediated endocytosis in exosome biogenesis. Concurrently, tubular carriers, a complex comprising MICAL-like protein 1 (MICAL-L1) and syndapin 2, a Bin/amphiphysin/Rvs (BAR) domain protein that inserts into the EE bilayer structure and bends the membrane, are also involved in EE membrane budding [30].

Guanosine triphosphate (GTP)-binding Rab proteins play pivotal roles in the formation of EEs. Through constant binding and hydrolysis of GTP, Rab can regulate the rate of vesicle fusion [31, 32]. Rab proteins are commonly used as specific markers for EEs and late endosomes, with Rab5a-labeled EEs and Rab7-labeled LEs [33]. This process involves the dissociation of Rab from guanosine diphosphate (GDP) and the binding of guanosine triphosphate (GTP) [32]. This process triggers a conformational change in Rab, rendering it capable of binding to vesicle surface proteins. GTP is hydrolyzed to GDP upon vesicle fusion and separated from Rab [32, 34]. It can thus be hypothesized that the binding, substitution, and hydrolysis of Rab with GDP and GTP can regulate the process of vesicle fusion in its entirety. As expected, Rab5 knockdown has been reported to decrease exosome excretion in triple-negative breast cancer cells [35].

## Regulation of the inward budding of MVBs

The second step of exosome biogenesis is the inward budding of endosomes and ILV formation, and multiple mechanisms are involved in regulating this step [36]. ESCRT complexes play crucial roles in mediating inward budding. In addition, several other pathways or proteins, including neutral sphingomyelinase 2 (nSMase2), caveolin-1, flotillins, cholesterol, and tetraspanins, also participate in the budding of the endosome membrane [37], the second step of exosomal biogenesis (Fig. 1c).

In ESCRT-dependent mechanisms, five proteins, including ESCRT-0, ESCRT-I, ESCRT-II, ESCRT-III, and VPS4-VTA1, act in a stepwise manner [38]. In detail, ESCRT-0 is capable of recognizing and segmenting PMs labeled by ubiquitination, the ESCRT-I clusters ubiquitinated transmembrane cargoes in the microdomains of the MVB endosomal membrane, and ESCRT-II prompts the endosomal membrane to invaginate cargos and form the initial budding body [39]. ESCRT-III subcomplexes at the neck of the budding body subsequently cause shearing of the budding body, which releases ILVs into the endosomal lumen to form the MVB [40, 41]. Finally, the Vps4/Vta1 complex depolymerizes the polymerized ESCRT-III, with hydrolyzed ATP providing the energy for recycling [38, 42]. Syndecan heparan sulfate proteoglycans and their cytoplasmic adaptor, syntenin, control the formation of exosomes. Syntenin interacts directly with ALG-2-interacting protein-X (Alix) throughLYPX(n)L motifs, similar to retroviral proteins, and supports ILV formation. Syndecan-Syntenin-Alix axis-mediated exosome biogenesis is ESCRT-dependent because silencing of the ESCRT-III component Chmp2a and its recycling component Vps4 markedly reduces the level of exosomal release.

On the other hand, researchers have knocked out the ESCRT complex but still found that exosomes with CD63 markers are released [43], which suggests that exosomes can also be generated in an ESCRT-independent manner. Sphingolipid ceramide generation at the PM and in the endosomal system is vital for EV formation [43, 44]. Ceramide possesses a distinctive conical molecular structure. Once inserted into the membrane, it alters the membrane curvature of subdomains on the endosomal membrane, thus facilitating ILV formation [43]. Moreover, ceramide participates in the formation of membrane microdomains, such as lipid rafts. These microdomains function as platforms for the aggregation and interaction of proteins and lipids associated with exosome biogenesis. For example, research has indicated that upon binding to fatty acids, endothelial cell CD36 enhances the secretion of exosomes by promoting the generation of ceramides [45]. After generation in the endoplasmic reticulum, ceramide is primarily transported to membrane structures such as the Golgi apparatus and MVBs via ceramide transfer protein (CERT) [46, 47]. Specifically, CERT can transport ceramide to early endosomes, where it induces membrane invagination, leading to the formation of ILVs. Additionally, through its interaction with tumor susceptibility gene 101 (TSG101), CERT couples ceramide transport to the ESCRT-dependent exosome biogenesis pathway, thereby initiating the exosome-formation cascade [48, 49]. Moreover, the nSMase2-ceramide pathway has been demonstrated to drive ILV formation and cargo sorting [43]. nSMase2 is a key enzyme that converts sphingomyelin (SM) into ceramide. GW4869, a well-characterized inhibitor of nSMase2, has been widely recognized as a crucial approach for effectively suppressing exosome biogenesis [43, 50]. In addition, other enzymes involved in SM metabolism, such as SM synthases 1 and 2, which convert ceramide to SM at the trans-Golgi and PM, respectively, to regulate exosome formation [51, 52]. Caveolin-1, as mentioned above, has been demonstrated to play a role in the process of EEs. It has been shown to bind to cholesterol in the PM, acting as a scaffold for lipid and protein assembly, initiating the process of membrane outgrowth at the PM and mediating caveolae-dependent endocytosis [53]. This process has been demonstrated to influence the diameter of the ILVs. For example, caveolin-1-deficient fibroblasts can produce compact and dense small-diameter ILVs [54]. Moreover, ubiquitinated caveolin-1 can be sorted into MVBs, which centrally regulate the ILV sorting of extracellular matrix (ECM) cargos [55–57].

Flotillins are membrane scaffolding proteins that drive lipid raft-dependent ILV formation, thereby regulating exosome production [58]. Rab31 drives the membrane outgrowth of MVBs to form ILVs by binding to flotillin proteins in lipid raft microdomains, which facilitates the fusion of MVBs and the PM, thus promoting exosome secretion [59].

In addition to flotillins, cholesterol plays an essential role in the formation of ILVs [44]. As a critical lipid raft component, cholesterol forms ordered, tightly packed membrane microdomains. Lipid rafts provide specialized platforms that facilitate the aggregation of proteins and lipids essential for ILV formation [46, 48]. Evidence suggests that cholesterol is instrumental in recruiting the ESCRT machinery, promoting ESCRT-mediated membrane budding, and forming MVBs [60]. Moreover, cholesterol has been implicated in regulating late endosome motility [47, 61] Specifically, cholesterol in late endosomes is recognized by oxysterol-binding protein-related protein 1 L (ORP1L), which governs the movement of endosomes along microtubules, supporting its critical role in exosome biogenesis [12, 47]. Cholesterol has also been shown to promote exosome secretion in oligodendrocytes and Huh7 hepatocellular carcinoma cells [49, 62]. The accumulation of cholesterol is associated with increased secretion of flotillin-containing exosomes [51], whereas the inhibition of nSMase2 impedes exosome production [63]. Recent studies have demonstrated that nicotinamide adenine dinucleotide phosphate generated through mitochondrial fatty acid oxidation drives cholesterol synthesis, promoting the biogenesis and release of exosomes in hepatic stellate cells [52]. Collectively, these findings underscore the multifaceted role of cholesterol in the regulation of ILV and exosome formation.

In addition, the four-transmembrane protein (tetraspanin) has also been demonstrated to participate in the ESCRT-independent pathway of ILV production. The tetraspanins include CD151, CD82, CD81, CD63, CD9, Tspan9 and Tspan7. Among these proteins, CD9, CD81, and CD63 are the principal components of vesicles and thus can be used as classical markers of exosomes [41, 64]. The tetraspanin family of membrane scaffolds assembles proteins into microstructural domains to control signaling and various cellular activities [65]. CD63 can couple apolipoprotein E (ApoE) to facilitate ILV formation during melanogenesis [66]. Low concentrations of tetraspanin-6 can interact with syntenin to promote ILV sorting in cells and the secretion of amyloid precursor proteins.

## Regulation of MVB fusion with the PM

After maturation, MVBs translocate intracellularly to the PM, where they ultimately fuse with the PM [67, 68]. This process releases ILVs into the extracellular space, where they form exosomes [69, 70], which is the third step in exosome formation (Fig. 1d).

Most intracellular transport pathways are subject to regulation by Rab family small GTPases, which function as molecular switches by cycling between a GTP-bound active state and a GDP-bound inactive state [71]. Approximately 60 different Rabs have been identified in mammals; these Rabs exhibit distinct intracellular localizations and regulate different steps of intracellular membrane transport [72]. In addition, other proteins in the Rab family have been shown to function in regulating exosome release. Polarized Madin‒Darby canine kidney epithelial cells asymmetrically release two different types of exosomes: one from the apical PM (apical exosomes) and the other from the basolateral PM (basolateral exosomes). Rab27 and Rab37 have been shown to regulate apical exosome release, whereas Rab39 has been shown to regulate basolateral exosome release [73].

In addition to the Rab family, lysosome-associated protein transmembrane 4B (LAPTM4B), heat shock protein 90 (HSP90), and endoplasmic reticulum (ER)-endosome contact sites promote MVB transport to the PM and increase exosome secretion [74–76]. Cytoskeletal proteins are a class of structural proteins that form a network structure within the cell comprising microfilaments, intermediate filaments, and microtubules [77]. Cytoskeletal proteins are also involved in the PM transport of MVBs. During MVB movement and secretion, the role of actin is twofold: first, it provides structural support within the cell, and second, it regulates the directional transport of vesicles [78]. Furthermore, cortactin is pivotal in regulating MVB movement and its attachment to the PM. This attachment increases the number of PM docking sites by stabilizing the branching actin structure [79]. In addition, cortactin has been found to regulate exosome release by binding to branching actin filaments and seven-subunit actin-related proteins-2/3 (ARP2/3). The overexpression of cortactin in cancer cells has been shown to increase exosome secretion [80].

## Regulation of MVB fusion with lysosomes

After MVB maturation, it can fuse with the PM and release exosomes. In contrast, if MVB is not released through the PM after maturation but is phagocytosed by lysosomes, it will be degraded, thus inhibiting exosome production (Fig. 1e).

Multiple pathways regulate MVB lysosomal degradation. As mentioned above, high concentrations of tetraspanin-6 can bind to syntenin/syndecan as a trimer, targeting syntenin to lysosomes to inhibit the formation of exosomes [81]. Our laboratory’s previous study identified neddylated Coro1a as a novel regulator of exosome biogenesis that promotes Rab7-mediated lysosomal targeting, and inhibition of Coro1a neddylation can increase exosome production [40]. Aldo-keto reductase family 1 member B (AKR1B) promotes lysosomal degradation of MVB by negatively regulating the expression of transcription factor EB (TFEB) and Rab7a [82]. In addition, ISGylation of the MVB protein TSG101 controls ubiquitin-like modifications associated with exosome release. The binding of ISG15 to TSG101 triggers the colocalization of MVBs with lysosomes and promotes the aggregation and degradation of MVBs, leading to reduced exosome secretion [83].

## Regulation of exosome cargo sorting

**Fig. 2:**
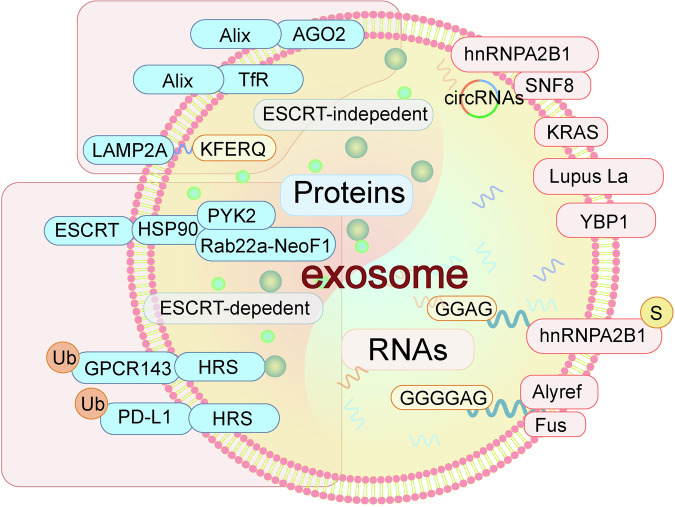
Regulation of exosome cargo sorting. Proteins are sorted into exosomes through ESCRT-dependent mechanisms, such as ubiquitinated GPCR143 and PD-L1, which are sorted to exosomes after being recognized by HRS. Tetraspanis and Rab22a-NeoF1 can be recognized by ESCRT complexes and are thus sorted into exosomes. ESCRT-independent pathways involve LAMP2A-dependent exosomal sorting of proteins with the KFERQ motif. Alix-associated Ago2 or TfR are sorted to exosomes via an ESCRT-independent pathway. Proteins, including SNF8, KRAS, Lupus La, and YBP1, are involved in miRNA sorting into exosomes. RBPs hnRNPA2B1, Alyref, and Fus facilitate the exosomal sorting of miRNAs by recognizing specific motifs in miRNAs

Exosomes are known to carry a variety of substances, including proteins, nucleic acids, lipids, and metabolites [84]. In addition to cell-specific cargos, exosomes possess several common markers. For example, proteins such as TSG101, HSP70, CD9, CD63, and CD81 are popularly used as specific biomarkers for exosome analysis. Exosomal proteins strongly affect exosome functions. Tetraspanins, such as CD9, CD63, CD81, and CD82, assist in cell penetration, invasion, and fusion processes [73]. HSP70 and HSP90 are involved in antigen presentation and binding [85]. MVB formation-associated proteins, such as Alix, lipid raft markers, and TSG101, participate in the biogenesis and release of exosomes [86]. Additionally, nucleic acids within exosomes play a critical role in cellular communication, with mRNAs or miRNAs incorporated into the membrane during exosome formation and capable of altering gene expression in recipient cells. Among these nucleic acids, miRNAs are the most abundant and influence various biological processes, including exocytosis, hematopoiesis, angiogenesis, and intercellular communication [87–89]. During the endosome inward budding process, many components in the cytoplasm can be passively sorted into exosomes. However, in the ESCRT-dependent exosome formation pathway, ESCRT-0 recognizes and selectively sorts ubiquitinated proteins into exosomes. In addition, many miRNAs can be sorted into exosomes by coupling with RNA-binding proteins (RBPs). Whether miRNA sorting is selective is determined by the exosomal selectivity of RBPs. Here, we introduce the selective sorting mechanism of proteins and miRNAs (Fig. 2).

## Exosome sorting of proteins

Proteins can be sorted into exosomes for cell-to-cell transport via ESCRT-dependent or ESCRT-independent forms. ESCRT is involved in membrane budding and exosome biogenesis, recognizing ubiquitinated proteins and sorting them into invaginated endosomal membranes for protein-specific selection [90]. For example, ubiquitinated G-protein coupled receptor 143 (GPCR143) can interact with HRS (ESCRT-0 subunit), facilitating its binding to transporter proteins and subsequent selective sorting of proteins into exosomes [91]. Our recent publication demonstrated that UBE4A-mediated ubiquitination of PD-L1 increases the exosomal sorting of PD-L1 after being recognized by HRS, leading to resistance to anti-PD-1 therapy [92]. In addition, proteins can be sorted into exosomes by direct binding to ESCRT components. Alix recruits ESCRT-III to LEs through interactions with lysophosphatidic acid (LBPA), potentially regulating the sorting of tetraspanins into exosomes [41]. HSP90 can directly interact with the ESCRT-I subunit TSG101 [93]. The Rab22a-NeoF1 fusion protein and its binding partner, PYK2, can be sorted into exosomes by interacting with HSP90 in the ESCRT-dependent pathway [94]. Proteins containing a KFERQ motif pentapeptide are loaded into a subpopulation of exosomes in a process dependent on the membrane protein LAMP2A, which is dependent on HSC70, CD63, Alix, Syntenin-1, Rab31, and ceramides rather than the ESCRT machinery [95]. Argonaute 2 (Ago2) can be associated with Alix and can thus be sorted into exosomes [96]. Similarly, the transferrin receptor (TfR) is sorted into exosomes through interactions with Alix [97].

## Exosome sorting of RNAs

Lötvall J. first discovered that exosomes naturally carry miRNAs and that the selective packaging of specific RNAs into exosomes is affected by various sorting mechanisms [16, 98]. Two articles showed that SNF8 (ESCRT-II subunit) and hnRNPA2B1 mediate the sorting of circular RNAs (circRNAs), including circRHOBTB3 and circNEIL3, respectively [99, 100]. In addition, KRAS, hnRNPA2B1, Alyref, Fus, Lupus La protein, and Y-box protein I (YBP1) have been reported to mediate RNA sorting into exosomes [101–104]. The specific motifs in miRNAs are essential for their exosomal sorting. Sumoylated hnRNPA2B1 specifically binds exosomal miRNAs by recognizing GGAG motifs and controlling their loading into exosomes. Alyref and Fus are involved in the export of miRNAs with CGGGAG into exosomes. Since miRNAs are sorted into exosomes via RBPs, exosomal selectivity is determined by RBPs. If RBPs are selectively sorted to exosomes, then miRNAs should also be identified. Although few studies have investigated how RBPs are sorted into exosomes, RBP Ago2 is likely selectively sorted into exosomes because it can directly interact with Alix [96].

## The role of exosomes in immunopathology

**Fig. 3:**
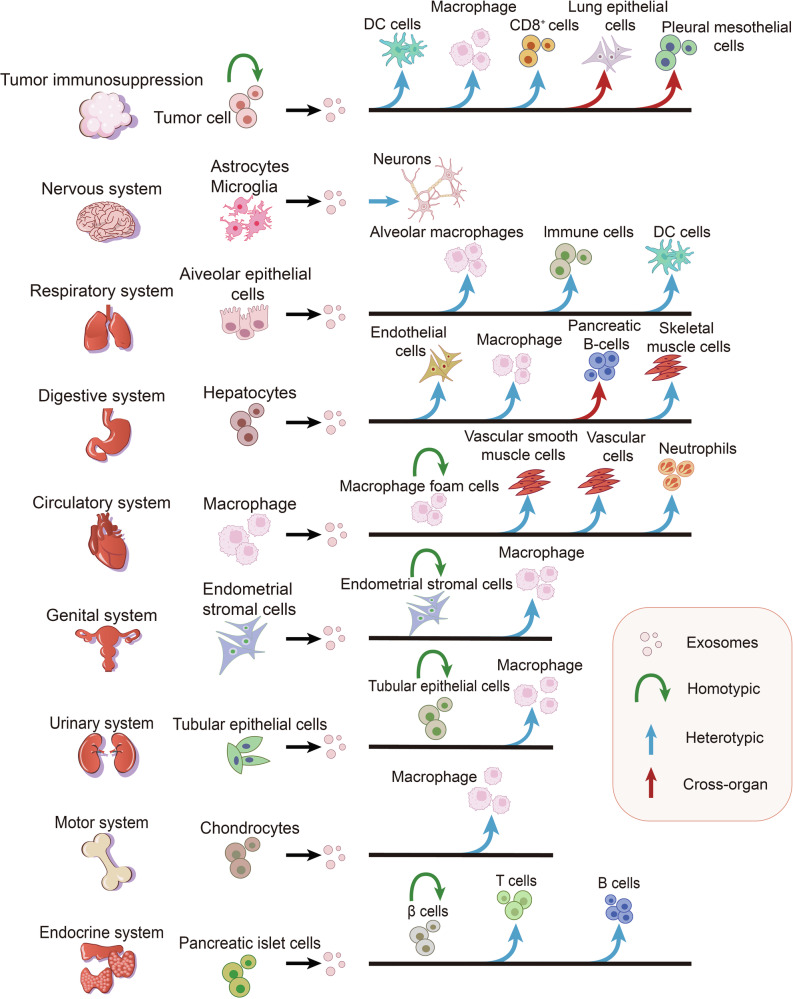
Schematic of exosome-mediated information communication between different types of cells. Exosomes mediate communication between different cells through homotypic, heterotypic, and cross-organ manners during tumor suppression and immunopathology of eight systems

The precise regulation of exosome biogenesis and cargo sorting ensures the efficient packaging of specific biomolecules, such as proteins, nucleic acids, and lipids, which endows exosomes with diverse biological functions. As key mediators of intercellular communication, exosomes can transfer functional cargo to recipient cells, modulating their behavior and facilitating dynamic interactions within the tumor and disease microenvironments. Exosomes can mediate communication between ‌homotypic (same-type) and heterotypic (different-type) cells. Remarkably, exosomes derived from cells in one organ can enter the peripheral circulation and reach distant organs, where they are internalized by recipient cells, enabling cross-organ communication. Therefore, we systematically review the immunopathological roles of exosomes in diseases across systems, focusing on three dimensions: homotypic cell interactions, heterotypic cell interactions, and cross-organ communication (Fig. 3). Table 1 lists the functions of exosomes in immunopathology.

**Table 1:** Exosomal sources, effector molecules, and functions in immunopathology

Source	Cargo	Acceptor cell	Function	Ref.
Tumor cells	miR-145	Macrophages	Induce macrophage polarization toward an M2 phenotype	[110]
Fleur Nagri protozoa	—		Promoting the production of the cytokine IL-8	[138]
Nociceptive neurons	miR-21		Driving a pro-inflammatory phenotype	[140]
Macrophages	—		Stimulating the pro-inflammatory response	[150]
Epithelial cells	—		Facilitate M1 macrophage polarization	[161–163]
Visceral AT	—		Promoting M1 macrophage Polarization	[166, 167]
Ectopic endometrial stromal cells	miR-146a-5p		Facilitating M2 macrophage polarization	[193]
Uterine cavity	miR-210-3p		Inducing M2 macrophage polarization	[196]
Uterine cavity	—		Decreasing the proportion of CD80^+^ macrophages	[197]
Syncytiotrophoblasts	5’-tRNA fragments		Causing inflammation in macrophages	[201]
Trophoblasts	—		Promoting macrophage to M1 polarization	[202]
Trophoblastic cells	miR-141		Inducing the formation of M1 macrophages	[203]
Renal tubular epithelial cells	*Ccl2* mRNA		Activating macrophages	[209]
Tubular epithelial cells	miR-23a		Promoting tubulointerstitial inflammation	[211]
Tubular epithelial cell	miR-19b-3p		Promoting M1 macrophage activation	[212]
Fibroblast-like synoviocytes	—		Promoting M1 macrophage polarization	[223]
Osteoarthritic chondrocytes	—		Increasing the production of mature IL-1β in macrophages	[224]
RA fibroblast-like synoviocytes	—		Stimulate macrophage migration	[225]
Serum	miR-6089		Activating macrophage	[226]
Synoviocytes	CDO1		Promoting M1 polarization	[227]
Adipocytes	miR-34a		Inhibiting M2 macrophage polarization	[240]
Adipocytes	miR-1224		Inhibits M2 macrophage polarization	[241]
Cardiomyocytes	miR-9-5p		Promotes M1 polarization	[174]
Alveolar epithelial cells	miR-92a-3p	Alveolar macrophages	Inducing pulmonary inflammation	[144]
Lung	miR-6238		Modulating neutrophil infiltration into the lung	[145]
Tumor cells	TGF-β1 and NKG2D ligands	NK cell	Reduce NKG2D expression	[112]
Tumor cells	—		Induce NK cell exhaustion	[113]
Endothelial cells	—	Neutrophils	Driving neutrophil reverse transendothelial migration to disseminate inflammation	[143]
*Escherichia coli*	IL-17A		Inducing neutrophilic inflammation	[159]
Cardiomyocyte	miR-9-5p		Inducing neutrophils to the N1 phenotype	[182]
Macrophages	miR-146a		Promoting NET formation	[190]
Endothelial cells	—	Monocytes	Promoting monocytes into pro-inflammatory macrophages	[142]
Nasal lavage fluid/ respiratory tract/Alveolar macrophages	—	Monocytes NKs Neutrophils	Promoting inflammation via TLR signaling	[151–153]
Tumor cells	—	DCs	Impairing their differentiation and maturation	[105]
Tumor cells	Tumor antigens		Induced antitumor-specific CD8^+^ T cell responses	[123]
Ascites	Mart1		Triggered tumor-specific T-cell responses	[124]
Alveolar epithelial cells	—		Facilitating activation of monocyte-derived DCs	[154]
Tumor cells	—	Immature DCs	Hindering their maturation through TGF-β1	[107]
Tumor cells	—	Myeloid precursors	Inhibiting the differentiation of myeloid precursors into DC	[106]
Tumor tissue	—	T cells	Through decreased T-cell receptor signaling	[115]
B cells	—		Impairing CD8^+^ T cell responses	[120]
Plasma samples from NPC patients	—		Inhibiting T cell proliferation	[116]
B cells	MHC class II molecules		Activating T cells	[15]
β cells	IA-2 GAD65		Enhancing antigen presentation, T cell activation	[232]
Trophoblastic cells	miR-141	Jurkat T cells	Reducing Jurkat T cell proliferation	[205]
M1 macrophages	—	T_H_ cells	Worsening experimental autoimmune neuritis by enhancing T_H_1 and T_H_17 responses	[139]
T lymphocytes	miR-142-3p	Treg cells	Impairing Treg cell function	[237]
Tumor cells	—		Promoting Treg cell expansion	[117, 118]
Primary islet cells	—	T and B cells	Activate T and B cells	[235]
Tumor cells	—	B cells	Producing pro-tumorigenic IgG	[119]
Astrocyte	Components	Neurons	Damaging neurons	[129]
Macrophages	miR-146a	Microglial	Hindering Aβ clearance	[130]
Macrophages	miR-155-5p	Glial	Inducing inflammatory responses	[133]
Activated monocytes Activated macrophages	Neurotoxic cargo	Neurons Astrocytes	Promoting cellular damage	[134]
Microglia	α-Syn	Neuron	Neuronal damage	[135]
Fleur Nagri protozoa	—	Glial Microglial	Triggering a pro-inflammatory immune response	[137]
Microglia	Viral RNA	Bystander CNS cells	Promoting an inflammatory immune response	[141]
Tumor cells	—	Pleural mesothelial cells	Enhancing DC infiltration into lung tumors	[128]
Trophoblasts	DDIT4	Endothelial cells	Triggering the inflammatory response	[204]
Macrophages	miR-21-3p	Vascular smooth muscle cells	Enhancing vascular smooth muscle cell migration and proliferation	[186]
Macrophages	CD5L	Vascular smooth muscle cells	Mediating lipid metabolism-induced inflammatory damage	[188]
Macrophages	Circ_100696	Vascular smooth muscle cells	Enhancing vascular smooth muscle cell proliferation and migration	[189]
Liver	—	Skeletal muscle and the pancreas	Enhancing glucose effectiveness and insulin secretion	[178]
Peritoneal macrophages	LncRNA CHL1-AS1	Endometrial stromal cells	Promoting cell proliferation, migration, and invasion and inhibiting apoptosis,	[198]
Macrophages	—	Renal cell, Neutrophils	Pro-inflammatory effect and enhancing renal cell IL-8 production and the migration of neutrophils	[214, 215]
Macrophage	miR-155	Mesenchymal stem cells	Inhibiting the osteogenic differentiation of mesenchymal stem cells	[219]
Neoplastic mast cells	miR-30a miR-23a	Osteoblast	Blocking osteoblast differentiation	[221]
β cells	—	β cell	promote a pro-inflammatory islet transcriptome	[229]
T lymphocytes	miR-142-3p miR-142-5p miR-155	β cells	Promoting apoptosis	[244]
Macrophages	miR-210-3p,	Adipocytes	Influencing insulin sensitivity	[243]
Alveolar epithelial cells	Tenascin-C	—	Exacerbating ALI	[148]
Macrophages	miR-223	—	Inducing intestinal barrier dysfunction	[168]
Neutrophils	PAD4	—	Impairing intestinal barrier integrity	[170]
Macrophages	miR-16-5p	—	Promoting the progression of AS	[187]
Macrophages	miR-27-3p	—	Activating inflammation	[242]

## Exosomes in tumor immunosuppression

Tumor immunity plays a pivotal role in identifying and eliminating tumor cells, but cancer cells often develop sophisticated strategies to evade immune surveillance. The tumor microenvironment (TME), which is composed of tumor cells, immune cells, stromal cells, and ECM components, profoundly impacts both tumor growth and immune responses. Interactions among various cells within the TME can either promote tumor progression by dampening immune activity or control cancer by promoting immune responses. The role of exosomes in tumor immunity is gaining increasing attention, as tumor-derived exosomes (TEXs) profoundly modulate a wide range of immune cells through heterotypic and cross-organ communication, shaping the tumor immune landscape and influencing tumor progression.

In terms of heterotypic cell communication, almost every type of immune cell can be influenced by TEXs. As principal antigen-presenting cells, dendritic cells (DCs) are crucial for inducing primary T-cell responses and enhancing antitumor immunity. However, tumor-derived exosomes (TEXs) can disrupt DC function by impairing DC differentiation and maturation [105]. On the one hand, TEXs inhibit the differentiation of myeloid precursors in the bone marrow into DCs through the induction of IL-6 [106]. On the other hand, TEXs can be taken up by immature DCs, further hindering their maturation through TGF-β1 [107]. Macrophages are more susceptible to modification in the tumor microenvironment [108]. Upon stimulation by TEXs, macrophages release cytokines closely associated with tumor invasion and metastasis [109]. TEXs containing miR-145 can also induce macrophage polarization toward the M2 phenotype by downregulating *histone deacetylase 11*, which supports tumor progression [110]. Natural killer (NK) cells, a type of cytotoxic lymphocyte, play an essential role in antitumor responses and function independently of antigen-dependent mechanisms [111]. TEXs that deliver TGF-β1 and NKG2D ligands can reduce the expression of NKG2D, a key receptor for NK cell cytotoxicity [112]. Additionally, the cargo carried by TEXs can decrease the production of IFN-γ and TNF by NK cells or induce NK cell exhaustion by increasing the expression of TIM-3 [113]. The tumor-associated macrophage-derived exosomal long intergenic noncoding RNA (ncRNA) LINC01232 induces immune escape in glioma by downregulating tumor MHC-I [114].

Both CD4^+^ and CD8^+^ T cells are critical for antitumor immunity. TEXs also impair the quality and function of CD8^+^ T cells through decreased T-cell receptor signaling and diminished cytokine and granzyme B secretion [115]. More significantly, TEXs carry PD-L1 on their surface. Stimulation with IFN-γ increases the amount of PD-L1 on these vesicles, suppressing the function of CD8^+^ T cells and facilitating tumor growth. Our group also reported that PD-L1-carrying TEXs can induce resistance to anti-PD-L1 therapy by decoying anti-PD-L1. TEXs can inhibit T-cell proliferation and differentiation into type 1T helper (T_H_1) and T_H_17 cells while promoting regulatory T (Treg) cell generation [116]. Furthermore, TEXs can promote Treg cell expansion, enhancing their immunosuppressive function by converting CD4^+^CD25^−^ T cells into Tregs or downregulating PTEN [117, 118]. TEXs also affect B cells by stimulating them to produce protumorigenic IgG, thereby promoting tumor progression [119]. In addition to TEXs, immune cell-derived exosomes have also been demonstrated to be immunosuppressive. We reported that CD19^+^ exosomes from B cells, through CD39 and CD73 vesicle-incorporated proteins, hydrolyzed ATP from chemotherapy-treated tumor cells into adenosine, thus impairing CD8^+^ T-cell responses [120].

TEX-mediated signaling is not confined to the primary tumor site. TEX-mediated cross-organ information communication plays a crucial role in tumor metastasis. Integrins on TEXs determine their organotropism and promote the expression of the proinflammatory gene *S100* in metastatic niches through integrin signaling. This process mediates organ-specific tumor metastasis and represents a classic example of cross-organ immune regulation [121]. Additionally, RNAs carried by TEXs activate TLR3 signaling in lung epithelial cells, facilitating the recruitment of inflammatory neutrophils to promote pulmonary metastasis [122]. These findings suggest that TEXs may foster an inflammatory microenvironment in metastatic organs, indirectly suppressing antitumor immunity and promoting tumor progression.

## Exosomes in tumor immune activation

Heterotypic cell communication involves TEXs transferring signals to immune cells. As early as 1996, Raposo reported that B-cell-derived exosomes containing MHC class II molecules could induce antigen specific, MHC class II-restricted T-cell responses in vitro. However, this study did not explore whether such exosomes could activate T cells in vivo [15]. Zitvogel made critical contributions to exosome-based cancer immunotherapy. She first demonstrated that TEXs could transfer tumor antigens to DCs and that subsequent infusion of these DCs induced antitumor-specific CD8^+^ T-cell responses [123]. Additionally, her team discovered that exosomes isolated from malignant effusions of cancer patients carried the Mart1 tumor antigen, which could be delivered to DCs. These DCs then trigger tumor-specific T-cell responses in the peripheral blood of patients [124]. In a phase I clinical trial, autologous DC-derived exosomes loaded with MAGE3 melanoma antigenic peptides were successfully generated. Vaccination with these exosomes induced a partial response in one patient [125]. Similarly, studies have shown that DCs “pulsed” with TEXs suppress mouse hepatocellular carcinoma progression [126]. Notably, these studies relied on artificial manipulation of exosomes and failed to reflect the true biological functions of endogenous TEXs.

In contrast to cytokines or drugs, which typically exhibit unidirectional effects on tumors, TEXs carry diverse components inherited from parental cells, including proteins and genetic material, with a subset of these components demonstrating tumor-suppressive properties and others exerting tumor-promoting effects. Our research revealed that TEXs promote tumor metastasis by inducing IL-6 secretion from DCs; they simultaneously activate DCs and induce tumor antigen-specific CD8^+^ T-cell responses. Strikingly, IL-6 deletion converted TEXs into potent antitumor agents [127]. Furthermore, although TEXs accelerate pulmonary tumor progression, they also activate pleural mesothelial cells, increasing DC infiltration into lung tumors and partially stimulating antitumor immunity [128]. Overall, the role of TEXs in tumor progression likely depends on the dominant force (protumor vs. antitumor) within the in vivo microenvironment.

## Exosomal immunopathology in the nervous system

Research has fully demonstrated that exosomes are involved in the progression of central nervous system (CNS) disorders, especially neurodegenerative diseases. The immunopathology of exosomes in CNS diseases mainly manifests as neuroinflammation, leading to abnormal dysfunction of neurons.

## Exosomes in neurodegenerative diseases

Alzheimer’s disease (AD) is the leading cause of dementia, affecting ~50 million individuals globally. The disease is defined neuropathologically by the presence of amyloid plaques resulting from the extracellular aggregation of amyloid-beta (Aβ) peptides and neurofibrillary tangles within neurons. Exosomes play a pivotal role in AD progression by mediating information communication between heterotypic cells, which promotes neurotoxicity. Specifically, astrocyte-derived exosomes carry high levels of complement components, which may damage neurons during the late inflammatory phase of AD [129]. Additionally, miRNAs present in exosomes are involved in the clearance of Aβ. Elevated levels of miR-146a enhance microglial tolerance to Aβ and lipopolysaccharides (LPS), whereas inflammatory macrophages release circulating exosomes that further reinforce this tolerance, ultimately hindering Aβ clearance [130]. Parkinson’s disease (PD) is a prevalent neurodegenerative disease in which the formation of misfolded and aggregated α-synuclein (α-syn) is a key neuropathological hallmark [131]. In PD, exosomes contribute to neurodegeneration by facilitating inflammatory responses and delivering neurotoxic cargo between heterotypic cells, with M1 microglial activation playing a key role in regulating proinflammatory cytokines [132]. Moreover, inflamed macrophages have been identified as contributing factors in PD, as they release exosomes enriched with miR-155-5p, which is likely a critical inducer of inflammatory responses in glial cells [133]. Furthermore, the cargo carried by exosomes can drive proinflammatory phenomena. Exosomes containing α-syn derived from α-syn fibril-treated microglia contribute to neuronal damage by increasing the expression of TNF receptor superfamily member 10B (TNFRSF10B) and inducing a proinflammatory phenotype [134]. In addition to enabling local neuroimmune interactions, exosomes enable cross-organ communication. Exosomes released by activated monocytes and macrophages can cross the blood‒brain barrier and be internalized by neurons and astrocytes. These exosomes deliver neurotoxic cargo and promote cellular damage by mediating inflammatory cytokines such as IL-1β and TNF [135].

## Exosomes in other CNS diseases

Meningitis is characterized by inflammation of the meninges. It is typically diagnosed through abnormal white blood cells in the cerebrospinal fluid and specific clinical signs and symptoms [136]. Fleur Nagri protozoa, a pathogenic amoeba responsible for fatal brain infection and primary amoebic meningoencephalitis, have been shown to release exosomes (NfEXOs) that distinctly modulate inflammation. In a heterotypic cell transfer manner, NfEXOs are internalized by glial and microglia, triggering a proinflammatory immune response [137]. In addition to their effects on glial cells, NfEXOs also target macrophages, inducing responses by upregulating costimulatory molecules and promoting IL-8 production [138]. However, neither article specifies which components of the NfEXO are responsible for these effects. In addition, exosomes derived from M1 macrophages have been shown to exacerbate experimental autoimmune neuritis by enhancing Th1 and Th17 responses [139]. The cargo on exosomes that promotes immune responses has been identified. In neuropathic pain models, exosomes containing miR-21 from dorsal root ganglia nociceptive neurons have been shown to transfer this miRNA to macrophages, driving a proinflammatory phenotype and contributing to pain hypersensitivity [140]. Exosomes secreted by microglia during early Theiler’s murine encephalomyelitis virus infection contain viral RNA and can activate uninfected bystander CNS cells, promoting an inflammatory immune response [141].

## Exosomal immunopathology in the respiratory system

In the respiratory system, exosomes derived from various cell types, including epithelial and endothelial cells, play essential roles in immune modulation and inflammation regulation.

## Exosomes in acute lung injury/respiratory distress syndrome

Acute lung injury and respiratory distress syndrome (ALI/ARDS) are common life-threatening lung diseases associated with acute and severe inflammation. Numerous studies have shown that exosomes play a vital role in the progression of ALI/ARDS, primarily through heterotypic cell communication. Endothelial cell-derived exosomes promote monocyte differentiation into proinflammatory macrophages [142], or neutrophils reverse transendothelial migration to disseminate inflammation [143]. Exosomes can also explain LPS-induced inflammation. Exosomes derived from alveolar epithelial cells treated with LPS activate alveolar macrophages and induce pulmonary inflammation mediated by miR-92a-3p in ALI [144]. Many studies have shown that both miRNAs and proteins in exosomes contribute to promoting ALI/ARDS. MiR-6238 is upregulated in lung-derived exosomes in response to particulate matter exposure and can be internalized into alveolar macrophages and modulate neutrophil infiltration into the lung alveoli by targeting CXCL3 [145]. Exosomes can also carry different miRNAs to promote macrophage proliferation and inflammation [146, 147]. Tenascin-C is an immunomodulatory ECM protein. The release of tenascin-C-carrying exosomes from alveolar epithelial cells under unresolved ER stress exacerbates ALI by intensifying sepsis-associated inflammatory responses [148].

## Exosomes in asthma

Asthma is a chronic inflammatory disease of the airways with a complex pathophysiology. Exosomes contribute to the pathology and progression of asthma by triggering proinflammatory responses through both homotypic and heterotypic cell communication [149]. In terms of homotypic cell communication, exosomes derived from *M. pneumoniae*-infected macrophages can be taken up by uninfected macrophages and stimulate the proinflammatory response via the TLR2-NF-κB/JNK signaling pathways [150]. In the context of heterotypic cell communication, exosomes can exacerbate inflammation by modulating the activity and function of immune cells. Exosomes isolated from asthmatic donors can simulate monocytes, NKs, and neutrophils, specifically, neutrophilic inflammation via TLR signaling [151–153]. Exosomes released by cells in response to environmental stimuli may promote inflammation. Exosomes derived from house dust mite-stimulated alveolar epithelial cells facilitate the recruitment, proliferation, migration, and activation of monocyte-derived DCs in mice through the CNTN1‒Notch2 axis [154]. In addition to directly interacting with immune cells, exosomes can affect cell permeability. PM_2.5_-induced exosomes from plasma can reduce connexin levels, increase cell permeability, and promote the secretion of inflammatory factors in PM_2.5_-induced asthma [155]. Tenascin-C is present in high quantities in the airways of people with asthma. The release of tenascin-C and proinflammatory exosomes in the airway are relevant to the biology of virally induced asthma exacerbation [156]. Selective sorting of T_H_2 inhibitory miRNAs into airway-secreted exosomes and increased release into the airway after house dust mite exposure are involved in the pathogenesis of allergic airway inflammation [157].

## Exosomes in chronic obstructive pulmonary disease

Chronic obstructive pulmonary disease (COPD) is a devastating, irreversible pathology that affects millions of people worldwide. COPD is characterized by emphysema, bronchitis, and obstructive airway disease, which represent a group of inflammatory lung conditions. Recent studies have highlighted the crucial role of exosomes in the progression and pathophysiology of COPD [158]. For example, exosomes derived from *Escherichia coli* can induce emphysema through IL-17A-mediated neutrophilic inflammation [159]. Furthermore, elevated levels of circulating exosomes in patients with COPD have been linked to systemic inflammation [160]. Both PM_2.5_ and smoking can aggravate COPD. Several studies have shown that exosomes derived from epithelial cells facilitate M1 macrophage polarization in COPD [161–163]. In addition to mediating effector molecule transfer between heterotypic cells, exosomes can also carry Wnt5a and proinflammatory cytokines, delivering these molecules to various organs, as observed in the blood of COPD patients, thereby exacerbating disease progression [164], highlighting the broader cross-organ communication role of exosomes in promoting the pathogenesis of COPD.

## Exosomal immunopathology in the digestive system

Recent research has revealed the pivotal role of exosomes in modulating gut and liver health, influencing diseases such as colitis, liver fibrosis, nonalcoholic fatty liver disease (NAFLD), and even metabolic disorders.

## Exosomes in inflammatory bowel disease

Inflammatory bowel disease (IBD) is a chronic intestinal condition characterized by inflammation and dysbiosis of the intestinal microenvironment. Exosomes shed from sites of intestinal inflammation in patients exhibit proinflammatory mRNA and protein profiles distinct from those of healthy individuals, suggesting that exosomes play crucial roles in maintaining intestinal homeostasis, functioning in heterotypic cells, and cross-organ communication [165]. In the IBD intestinal microenvironment, macrophages are recruited. Exosomes produced in IBD mice or visceral adipose tissue (AT) promote the production of TNF by macrophages or M1 macrophage polarization [166, 167]. Macrophage-derived exosomal miR-223 plays a novel role in the development of dextran sulfate sodium salt (DSS)-induced colitis by inducing intestinal barrier dysfunction via the inhibition of transmembrane and immunoglobulin domain-containing protein 1 (TMIGD1) [168]. Gasdermin D (GSDMD)-mediated release of IL-1β-containing exosomes is involved in the pathogenesis of intestinal inflammation [169]. Neutrophil-derived exosomes carrying peptidyl arginine deiminase type 4 (PAD4) compromise the stability of creatine kinase, Mitochondrial 1 (CKMT1), disrupting mitochondrial homeostasis and impairing intestinal barrier integrity [170]. A large body of literature also reports on exosomes and IBD. Owing to limited space, we do not introduce them in detail here.

## Exosomes in NAFLD

NAFLD is the most common cause of chronic liver dysfunction. Evidence from mouse and human models indicates that exosomes are crucial in the development of NAFLD [171]. Exosomes play a pivotal role in sustaining inflammation through heterotypic cell communication. Hepatocyte-derived exosomes in fatty liver also activate oncogenic signaling pathways, including the NF-κB pathway, thereby driving inflammation [172, 173]. Notably, miR-9-5p, which is upregulated in NAFLD-associated exosomes, promotes M1 polarization of THP-1 macrophages, further exacerbating inflammatory responses [174]. In lipid-rich environments, exosomes intensify inflammation by mediating various pathological processes. Lipids stimulate death receptor 5 (DR5), triggering the release of hepatocyte-derived exosomes that induce an inflammatory phenotype in macrophages [175]. Integrin β1 on exosomes released from hepatocytes under lipotoxic stress mediates monocyte adhesion to liver sinusoidal endothelial cells, an essential step in hepatic inflammation [176]. Palmitate-induced exosomes are enriched in C16:0 ceramide, which is metabolized to sphingosine-1-phosphate (S1P), facilitating the recruitment of macrophages to the liver under lipotoxic conditions [177]. In addition, exosomes derived from NAFLD patients are involved in glucose metabolism via cross-organ communication. In response to hyperglycemia, elevated levels of liver-derived exosomes from NAFLD patients are released into the circulation, enhancing glucose effectiveness and insulin secretion through direct cross-organ communication with skeletal muscle and the pancreas, respectively [178].

## Exosomal immunopathology in the circulatory system

Cardiovascular diseases are the most prevalent cause of death and morbidity worldwide. Several pieces of experimental data have emphasized that exosomes are involved in the immunopathology of cardiovascular diseases.

## Exosomes in heart failure

Exosomal communication also impacts structural remodeling between homotypic cells. Inflammatory fibroblast progenitor cell-derived exosomes exacerbate cardiac fibrosis through the miR-21a-5p/ITGAV/Col1α signaling pathway [179]. In addition to structural remodeling, exosomal communication also plays a key role in immune modulation through heterotypic interactions. Exosomes released from hypertrophied cardiomyocytes harbor proteins such as HSP90 and IL-6, which target the STAT3 pathway, which is essential for inflammation progression and heart failure pathogenesis [180]. Exosomes derived from ischemia‒reperfusion transfer miR-155-5p to macrophages and enhance the inflammatory response by activating the JAK2/STAT1 pathway [181]. This finding points to a crucial mechanism by which damaged cardiomyocytes influence immune cell behavior and postinjury inflammation. Furthermore, miR-9-5p on cardiomyocyte-derived exosomes was identified as a mediator that induced neutrophils to acquire the N1 phenotype. MiR-9-5p can also activate the JAK2/STAT3 and NF-κB signaling pathways in neutrophils [182].

## Exosomes in atherogenesis

Atherosclerosis (AS) is a lipid-driven, progressive inflammatory disease characterized by excess cholesterol deposition in the arterial wall, which leads to a nonresolving immune response [183]. First, homotypic signaling within the macrophage population plays a role in the severity of AS. In *Apoe*^*−/−*^ mice, exosomes derived from bone marrow-derived macrophages exposed to high glucose resulted in increased atherosclerotic lesions, with a notable accumulation of macrophage foam cells [184]. Heterotypic exosomal communication is prominent in AS. For example, obesity is closely linked to the development of AS, with recent studies highlighting the role of exosomal miRNAs in its progression. Obesity-induced exosomal miR-27b-3p contributes to endothelial inflammation and promotes AS by suppressing PPARα [185]. Moreover, exosomal miRNAs have been shown to promote AS under various conditions. Exosomal miR-21-3p derived from nicotine-treated macrophages may accelerate the progression of AS by enhancing vascular smooth muscle cell migration and proliferation through the inhibition of PTEN [186]. Exosomes derived from macrophages that encapsulate miR-16-5p can downregulate the expression of Smad7, thus promoting the progression of AS [187]. These findings highlight the complex roles of exosomal miRNAs in driving AS under various pathological conditions. Additionally, research has shown that heterotypic exosomal proteins also contribute to AS. CD5L, an exosomal protein secreted by macrophages, promotes AS development through lipid metabolism-induced inflammatory damage to vascular smooth muscle cells [188]. When exposed to stimuli, macrophages can influence the AS process through distinct mechanisms. Circ_100696 on exosomes derived from low-density lipoprotein (LDL)-induced macrophages enhances vascular smooth muscle cell proliferation and migration [189]. Exosomal miR-146a from oxidized LDL-treated macrophages promotes the formation of neutrophil extracellular traps (NETs) by inducing oxidative stress, thus contributing to the progression of AS [190]. Furthermore, there is evidence for cross-organ exosomal signaling. Exosomes enriched with miR-30c-2-3p exacerbate neuroinflammation in AS plaques and enhance microglial inflammatory properties in vivo by inhibiting Smad2 [191].

## Exosomal immunopathology in the genital system

Within the reproductive tract, distinct cell types must engage in precisely controlled communication to support complex processes such as embryo development and pregnancy. Therefore, exosomes naturally mediate information communication among various reproductive tract cells.

## Exosomes in endometriosis

Endometriosis is a gynecological disorder characterized by the growth of endometrium-like tissue within and beyond the pelvic cavity [192]. Exosomes are increasingly recognized as key mediators in their progression, primarily through heterotypic cell communication. Exosomes contribute to the progression of endometriosis by promoting M2 macrophage polarization. miR-146a-5p, present on exosomes derived from ectopic endometrial stromal cells, facilitates M2 macrophage polarization by targeting TNF receptor-associated factor 6 (TRAF6), which subsequently supports the development of endometriosis [193]. Similarly, exosomes from endometriotic tissue can enhance M2 macrophage polarization, promoting lesion development in mice [194]. This polarization is further amplified by miR-301a-3p, which is enriched in exosomes from endometriosis patients and promotes M2 differentiation via Arg-1 and PI3K upregulation and PTEN inhibition [195]. Additionally, miR-210-3p, present on exosomes from the uterine cavity, induces M2 macrophage polarization by modulating ATP5D expression, thereby promoting endometriosis progression [196]. Exosomes can also influence the number of macrophages; for example, exosomes from the uterine cavity in patients with endometriosis decrease the proportion of CD80^+^ macrophages by inhibiting the JNK signaling pathway, remodeling the immunological microenvironment in a way that favors the progression of endometriosis [197]. Furthermore, long noncoding RNAs (lncRNAs) on exosomes have been implicated in the development of endometriosis. The lncRNA CHL1-AS1, derived from peritoneal macrophages, is transferred via exosomes to ectopic endometrial stromal cells, where it promotes cell proliferation, migration, and invasion and inhibits apoptosis, thus facilitating the progression of endometriosis through the miR-610/MDM2 axis [198]. In addition, exosomal cross-organ communication contributes to endometriosis-related infertility. Exosomes from the vaginal environment of females with endometriosis contribute to infertility by promoting a Th17/Treg cell imbalance and suppressing sperm activity [199].

## Exosomes in eclampsia

Eclampsia is one of the most severe pregnancy complications and a leading cause of mortality among pregnant and postpartum women [200]. Recent studies have shown that exosomes contribute to the development of eclampsia through heterotypic cell communication. Exosomes are involved in manipulating macrophages in the process of eclampsia. 5’-tRNA fragments on syncytiotrophoblast exosomes in preeclamptic patients induce the production of 5’-tRF-Glu-CTC, which causes inflammation in macrophages [201]. Trophoblast-derived exosomes from women with preeclampsia promote preeclampsia by promoting M1 polarization in macrophages [202]. A hypoxic environment can upregulate miR-141 expression in the exosomes of HTR8-/Svneo cells (trophoblastic cells), which can be taken up by macrophages and induce the formation of M1 macrophages, which can lead to the development of preeclampsia [203]. In addition, exosomes destroy the function of endothelial cells to promote eclampsia. *An* in vitro study revealed that exosomes isolated from oxidative stress trophoblasts increase DNA damage-inducible transcript 4 (DDIT4) expression levels to trigger the inflammatory response to pyroptosis in endothelial cells through interactions with protein disulfide isomerase family a member 4 (PDIA4), which exacerbates preeclampsia [204]. MiR-141 is highly expressed on exosomes derived from trophoblastic cells in preeclamptic patients and can reduce Jurkat T-cell proliferation. These findings suggest their potential role in immune regulation [205].

## Exosomal immunopathology in the urinary system

Growing evidence highlights the pivotal role of exosomes in kidney physiology and pathology. Urinary exosomes facilitate communication between glomerular and tubular cells and among different tubular segments, whereas circulating exosomes promote interorgan communication and contribute to the progression of kidney damage and inflammation [206].

## Exosomes in acute kidney injury

Recent studies have highlighted the role of exosomes as mediators of intercellular communication in the progression of AKI, particularly through heterotypic signaling within the kidney. In acute kidney injury, exosomes secreted by renal cells accumulate in the urine, thus becoming a potential resource for disease treatment [207]. Exosomes secreted by epithelial cells are involved in the development of kidney diseases [208]. Exosomes carrying *Ccl2* mRNA derived from albumin-stimulated renal tubular epithelial cells can activate macrophages and induce renal tubular interstitial inflammation [209]. TGF-β1 carried by exosomes can activate fibroblasts when the resident parenchyma is injured [210]. HIF-1α-dependent release of miR-23a-enriched exosomes from hypoxic tubular epithelial cells activates macrophages to promote tubulointerstitial inflammation [211]. In addition, tubular epithelial cell-derived exosomes promote M1 macrophage activation via miR-19b-3p in LPS-induced acute kidney injury [212].

## Exosomes in chronic kidney diseases

Emerging evidence indicates that exosomes play a key role in the pathogenesis of CKD through both homotypic and heterotypic cell communication. In the homotypic context, in chronic kidney disease (CKD), exosomes from damaged tubular epithelial cells promote renal fibrosis [213]. In terms of heterotypic signaling, the exosomes produced by inflammatory cells under certain conditions can activate inflammation. Exosomes produced by macrophages stimulated with calcium oxalate monohydrate have a proinflammatory effect and enhance renal cell IL-8 production and neutrophil migration [214, 215]. High-phosphate-stimulated macrophage-derived exosomes promote vascular calcification via the let-7b-5p/TGFβR1 axis in CKD [216]. Moreover, exosomes from TNF-stimulated monocytes can induce inflammation and proteinuria in human podocytes [217], suggesting that exosomes are likely involved in CKD by regulating podocyte dysfunction.

## Exosomal immunopathology in the motor system

Maintaining the health of the motor system is essential for preserving a good quality of life, with bone‒muscle crosstalk playing a pivotal role in this process. This interaction is predominantly facilitated by exosomes released from various cell populations within muscle and bone.

## Exosomes in osteoporosis

Osteoporosis is a widespread chronic clinical condition characterized by an imbalance between osteoclast and osteoblast activity [218]. Exosomes play a significant role in mediating intercellular communication that influences bone homeostasis, particularly through transferring heterotypic signals between immune cells and bone-forming cells. Studies have demonstrated that immune cell-derived exosomes affect bone homeostasis. M1 macrophage-derived exosomes are enriched with miR-155, which suppresses the expression of BMP2, BMP9, and RUNX2, thereby inhibiting the osteogenic differentiation of mesenchymal stem cells [219]. M1 macrophage-derived exosomes can also exacerbate postmenopausal osteoporotic bone loss via the miRNA-98/DUSP1/JNK axis [220]. Exosomes from neoplastic mast cells in the serum of systemic mastocytosis patients deliver miR-30a and miR-23a, which block osteoblast differentiation and suppress the expression of RUNX2 and Smad1/5, which are essential drivers of osteogenesis [221].

## Exosomes in osteoarthritis

Osteoarthritis (OA) is a chronic joint disorder characterized by the gradual degeneration of articular cartilage [222]. Exosomes have emerged as critical mediators in the progression of OA by modulating immune responses, particularly through heterotypic cell communication. Exosomes influence macrophage polarization, thereby exacerbating OA. Inflammatory fibroblast-like synoviocyte-derived exosomes promote M1 macrophage polarization, which induces an OA-like phenotype in cocultured chondrocytes [223]. Substrate stiffness significantly enhances the ability of exosomes derived from maximal chondrocyte-mediated promotion of macrophage proliferation, migration, and inflammation, thereby contributing to the progression of OA [217]. Exosomes derived from osteoarthritic chondrocytes can activate the inflammasome and increase the production of mature IL-1β in macrophages. This elevated IL-1β production may worsen synovial inflammation and further accelerate the progression of OA [224].

## Exosomes in rheumatoid arthritis

Rheumatoid arthritis (RA) is a chronic inflammatory disease characterized by joint destruction and significant morbidity. Exosomes actively contribute to the progression of RA by modulating immune cell behavior through both heterotypic cell and cross-organ communication mechanisms. In the context of heterotypic cell communication, exosomes play a role in promoting RA by enhancing M1 macrophage polarization and T-cell differentiation. Exosomes derived from RA fibroblast-like synoviocytes stimulate macrophage migration through the PTX3 and PSMB5 axes [225]. Additionally, miR-6089, present on exosomes derived from the serum of RA patients, inhibits the LPS-induced proliferation and activation of macrophage-like THP-1 cells [226]. Exosomes from RA fibroblast-like synoviocytes also transfer cysteine dioxygenase I (CDO1), which promotes M1 polarization of macrophages, further advancing RA progression [227]. Moreover, cigarette smoke induces T_H_17 cells in the lymph node to package miR-132, which acts as a proinflammatory mediator by increasing osteoclastogenesis through the downregulation of cyclooxygenase-2 (COX2) in osteoclast precursor cells, thereby exacerbating RA [228], which exemplifies cross-organ communication.

## Exosomal immunopathology in the endocrine system

Exosomes are critical in the immunopathology of the endocrine system. Studies have shown that exosomes can accelerate endocrine system diseases by modulating innate and adaptive immune cells.

## Exosomes in diabetes mellitus

Diabetes mellitus, a systemic condition with a rapidly increasing global prevalence, is defined by persistent hyperglycemia caused by insulin resistance (IR) and/or impaired insulin secretion. Exosomes are increasingly recognized as critical mediators in the pathogenesis of both type 1 diabetes (T1D) and type 2 diabetes (T2D), influencing immune responses, metabolic regulation, and β-cell destruction through homotypic cells, heterotypic cells, and cross-organ communication. Homotypic cell communication is evident in that exosomes derived from proinflammatory β cells have been shown to induce β cell dysfunction and promote a proinflammatory islet transcriptome [229]. In terms of heterotypic cell communication, exosomes derived from adipocytes preferentially target circulating monocytes in vivo, enhancing macrophage activation and worsening IR [230, 231]. β-cell-derived exosomes also contain intracellular autoantigens, such as protein tyrosine phosphatase-like molecule IA-2 and glutamic acid decarboxylase 65 kDa isoform (GAD65), which increase antigen presentation, T-cell activation, and β-cell death [232]. Moreover, elevated levels of miRNAs in exosomes derived from T cells exacerbate cytokine overexpression, leading to pancreatic islet cell death [233, 234]. In the context of cross-organ communication, type 1 diabetes, a T-cell-mediated autoimmune condition, destroys insulin-producing β cells in the pancreas. In vivo, exosomes derived from primary islet cells can activate T and B cells in the spleen via the TLR4/MyD88 signaling pathway and induce the production of cytokines, including IL-6, IFN-γ, TNF, and monocyte chemotactic protein-1 (MCP-1), endogenously [235].

## Exosomes in the thyroid

Hypothyroidism occurs when the thyroid gland fails to produce sufficient thyroid hormones to meet the body’s needs. Compared with those in normal controls, the protein profiles of plasma exosomes in patients with autoimmune thyroid diseases are upregulated, suggesting that plasma exosomes may play a significant role in systemic immune imbalance in patients with autoimmune thyroid diseases [236]. Homotypic cell communication is involved in the autoimmune response within the thyroid microenvironment. For example, exosomal miR-142-3p from T lymphocytes within the tissue can impair Treg function and contribute to thyrocyte destruction, playing a role in the progression of Hashimoto’s thyroiditis [237]. In aging-associated hypothyroidism and acute thyroidectomy, cross-organ communication is evident, as liver-derived exosomes carrying ApoE4 cross the blood‒brain barrier and are taken up by neural cells. This process impacts cognition by activating the NLRP3 inflammasome and increasing cholesterol levels in neural cells [238]. Heterotypic exosomal signaling plays a pivotal role in promoting systemic immune imbalance. Exosomes derived from IFN-γ-treated thyroid cells activate DCs, which may increase the production of proinflammatory cytokines by CD4^+^ T lymphocytes, contributing to the development of autoimmune thyroiditis [239].

## Exosomes in obesity

Obesity is a complex metabolic condition characterized by excessive fat accumulation, leading to a chronic low-grade inflammatory state. Obesity disrupts insulin sensitivity and promotes adipose inflammation, with exosomes playing a critical role in these processes. Exosomes influence both macrophage polarization and T-cell function in obesity. Exosomes are emerging as crucial mediators of heterotypic cell communication in obesity, influencing immune responses and insulin sensitivity. For example, adipocyte-secreted exosomal miRNA-34a inhibits M2 macrophage polarization, promoting obesity-induced adipose inflammation [240]. Exosomal miR-1224, derived from adipocytes, also inhibits M2 macrophage polarization by targeting MSI2, further contributing to obesity-induced AT inflammation [241]. Additionally, miR-27-3p, which is highly expressed in exosomes from M1 macrophages, impairs mitophagy through the miR-27-3p‒Miro1 axis, which activates inflammation and drives the development of insulin resistance [242]. The AT microenvironment encourages AT macrophages to release exosomes containing miR-210-3p, which can be transferred to neighboring cells, influencing insulin sensitivity [243]. Furthermore, human T lymphocytes release exosomes containing miRNAs such as miR-142-3p, miR-142-5p, and miR-155. These exosomal miRNAs can be transferred in an active form to β cells, promoting apoptosis during autoimmune attack [244].

## Strategies to mitigate the pathological effects of exosomes

**Fig. 4:**
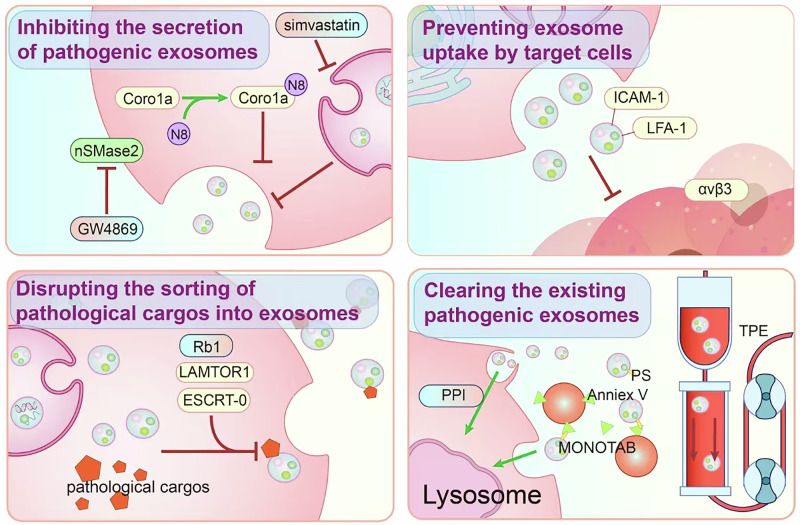
Strategies to mitigate the pathological effects of exosomes. Four strategies to prevent exosomal pathological effects. i) Reducing the generation of pathological exosomes; ii) inhibiting exosome uptake by target cells; iii) preventing the sorting of pathogenic cargos into exosomes; and iv) clearing existing pathological exosomes

Given their involvement in the pathological process of various diseases, exosomes are promising intervention targets for preventing and treating related diseases. We believe that four strategies exist to achieve this goal. First, reducing the generation of pathological exosomes, which blocks the pathogenic effect of exosomes from the source; second, inhibiting the uptake of exosomes by target cells, thereby weakening the pathological functions of exosomes; third, preventing pathogenic effector cargos from being sorted into exosomes, thereby downregulating the pathogenicity of exosomes; and fourth, eliminating existing pathogenic exosomes (Fig. 4).

## Inhibiting the secretion of pathogenic exosomes

Reducing exosome secretion involves targeting critical pathways and molecules that regulate their production. Inhibition of the migration of tumor cells can be achieved by preventing the intercellular exchange of M2 macrophage-derived exosomes related to caveolin-1 [245]. Rab5 is a small GTPase critical for EE trafficking. Rab5 knockdown reduces the production of exosomes and promotes macrophage polarization toward an antitumor phenotype, suggesting that Rab5 could serve as a potential therapeutic target for triple-negative breast cancer [35]. Knocking down the ESCRT-0 subunit HRS significantly reduces the expression of exosomes derived from head and neck squamous cell carcinoma and enhances the activation of CD8^+^ T cells [246]. GW4869, an nSMase2 inhibitor, inhibited 4T1 tumor growth in BALB/c mice by inhibiting TEX production [247]. The syndecan-syntenin-Alix pathway, regulated by phospholipase D2 (PLD2), promotes MVB formation. When human prostate cancer bone metastasis-derived C4-2B cell lines are treated with halopemide, a paninhibitor of PLD, the exosomes secreted by C4-2B cells lose their ability to stimulate osteoblasts, thereby inhibiting bone metastasis [248]. Cholesterol is another key player in MVB formation. The cholesterol synthesis inhibitor simvastatin reduces exosome release from cardiomyocytes, attenuating angiotensin II (AngII)-induced cardiac fibrosis, highlighting its potential role in modulating exosome release and mitigating cardiac fibrosis [249]. Furthermore, we revealed that neddylated Coro1a is a novel exosome biogenesis regulator that facilitates Rab7-mediated lysosomal targeting. Knockout of Coro1a or enhancement of Coro1a neddylation can effectively decrease TEX production, facilitating antitumor activation [40]. Lysosomal function can effectively influence exosome biogenesis [83]. PTEN can suppress tumor metastasis by enhancing lysosome acidification and reducing exosome release through a TFEB-dependent mechanism [250]. Rab27a benefits the PM transport of MVBs and the subsequent fusion of MVBs and the PM. Rab27a has been widely used to regulate exosome production. We found that TEXs are responsible for resistance to anti-PD-L1 therapy and that Rab27a knockout-mediated inhibition of TEX production can increase the sensitivity of tumors to anti-PD-L1 therapy [251]. The SNARE complex is proposed to mediate the fusion of MVBs with the PM. Deletion of VAMP-7 in 4T1 cells inhibits exosome secretion, significantly reducing tumor growth and lung metastasis [252].

## Preventing exosome uptake by target cells

Integrins and endocytic pathways both play crucial roles in promoting the absorption of exosomes by target cells. Blocking the absorption of pathological exosomes by target cells can help mitigate disease progression. Integrins such as αvβ3 are essential for exosome uptake, and inhibiting their activity markedly reduces exosome absorption [253]. Target cell receptors such as ICAM-1 and LFA-1 commonly bind exosomes. Blocking ICAM-1 on TEXs decreases their interaction with CD8^+^ T cells and mitigates the PD-L1-mediated immunosuppressive effects of TEXs [254]. Inhibiting endocytic pathways can also reduce the uptake of exosomes by cells. Moreover, chemical endocytosis inhibitors that target heparan sulfate proteoglycans, actin, tyrosine kinase, dynamin-2, sodium/proton exchangers, or PI3Ks have been shown to significantly decrease the internalization of BMSC-derived exosomes by multiple myeloma cells [255].

## Disrupting the sorting of pathological cargos into exosomes

The interventions mentioned above lack specificity for individual molecules, which may impact normal cellular functions. A better approach to reduce the number of pathological exosomes is to elucidate the mechanisms involved in cargo sorting into exosomes, as this could lead to the development of novel therapeutic strategies for disease treatment. Circ-0034880-enriched TEXs facilitate strong interactions between primary tumor cells and protumor macrophages, promoting the formation of the premetastatic niche and colorectal cancer liver metastasis. Rb1 effectively inhibits circ-0034880 loading in TEXs, reversing this phenomenon [256]. Exosomal miR-21 derived from tubular epithelial cells may accelerate the progression of renal fibrosis through the miR-21/PTEN/Akt pathway. Inhibition of epithelial exosomal miR-21 abolishes fibroblast activation in vitro [257]. Late endosomal/lysosomal adaptor and MAPK and MTOR activator 1 (LAMTOR1) facilitate PD-L1 lysosomal degradation by interacting with HRS, thereby reducing exosomal PD-L1. Using LAMTOR1 to design peptides enhances the efficacy of immunotherapy in lung cancer [258]. Our group recently discovered that tumor cell-derived MFGE8 specifically promotes PD-L1 sorting onto exosomes through the integrin signaling pathway. MFGE8-neutralizing antibodies can negate anti-PD-1 therapy resistance by preventing PD-L1 sorting onto exosomes [92].

## Clearing existing pathogenic exosomes

Recent advances have led to the development of several innovative strategies for exosome clearance. One such approach is modified nanoparticle with targeting binders (MONOTAB), a plug-and-play monofunctional degradation platform that can drag extracellular targets into lysosomes for degradation. Owing to the high levels of phosphatidylserine on the outer leaflet of exosomes, researchers have used Annexin V-coated nanoparticles to capture exosomes. MONOTAB subsequently mediates the degradation of exosomes by lysosomes through the inherent lysosome-targeting ability of these nanoparticles [259]. Orme et al. pioneered the use of therapeutic plasma exchange (TPE) to remove soluble PD-L1- and PD-L1-positive exosomes from the circulation in patients with malignant melanoma. Therapeutic plasma exchange (TPE) is a procedure in which blood is passed through an apheresis machine that separates plasma from cellular components. The removed plasma is discarded and replaced with a colloid solution, such as albumin. TPE effectively clears plasma-restricted substances, such as large antibodies and exosomes, that are too large for rapid diffusion. On average, each session removes ~65–70% of these noncellular, intravascular components [260]. Moreover, our group reported that proton pump inhibitor-induced macropinocytosis facilitates the clearance of immunosuppressive exosomes from tumor cells, thereby enhancing antitumor immunity [261].

## Perspectives and challenges

Cytokines are indispensable mediators of information exchange between different cells and are essential for regulating various physiological and pathological processes. Like cytokines, exosomes play a pivotal role in information exchange between cells. In addition, unlike cytokines, which are involved mainly in the functional regulation of neighboring cells, exosomes can cross-organly communicate and play a unique role in cross-organ functional regulation. Therefore, the role of exosomes in mediating intercellular communication is likely no less critical than that of cytokines; thus, their functional importance should not be underestimated. Elucidating the cross-organ regulatory function of exosomes, especially the cross-organ distribution pattern of exosomes in disease states, will expand our understanding of the pathogenesis of related diseases, thereby facilitating the identification of new therapeutic targets.

However, current exosome research faces numerous challenges due to ‌technical bottlenecks and a limited understanding‌. The stochastic nature of cargo loading into exosomes and the significant size variation of vesicles formed during MVB budding result in substantial heterogeneity‌, even among exosomes derived from the same cell type. This heterogeneity is the root cause of the lack of specific exosome biomarkers. In addition, while exosomes from different cell sources exhibit distinct functions, the functional implications of heterogeneity within exosomes of the same cellular origin remain unclear. The emergence of single-vesicle analysis techniques enables the identification of functional molecules on individual vesicles, offering a potential solution to these challenges. In the study of exosome biodistribution, current approaches rely primarily on labeling exosomes and tracking their postreintroduction distribution in vivo‌. However, this method likely differs significantly from the ‌native distribution of endogenously produced exosomes‌, posing a major challenge for real-time, precise tracking of endogenous exosomes. Additionally, the absence of ‌core universal biomarkers‌ makes absolute isolation of exosomes technically unachievable. Consequently, experimental results are inevitably confounded by contamination from other EVs and nonvesicular components‌. Moreover, many studies use exosomes isolated from heterogeneous tissues or organs, making it impossible to define their ‌cell source‌s. Notably, exosomes from distinct cell sources may perform similar functions if they carry shared effector molecules. Furthermore, in the disease treatment strategy of targeting exosomes, inhibiting the secretion of exosomes is likely to destroy the critical physiological functions of exosomes because exosomes secreted by normal cells are inevitably inhibited. Therefore, discovering how target cells uniquely take up exosomes in disease states and how pathogenic effector molecules are sorted explicitly into exosomes is more likely to lead to the development of disease-specific therapeutic targets. In summary, despite enormous challenges, exosomes have undoubtedly become a promising target for treating human diseases.
